# Language Experience Shapes Neural Grouping of Speech by Accent: EEG Evidence from Native, Second-Language, and Heritage Listeners

**DOI:** 10.3390/brainsci16020174

**Published:** 2026-01-31

**Authors:** Lauren L. Hong, Chao Han, Philip J. Monahan

**Affiliations:** 1 Department of Psychology, University of Toronto Scarborough, Toronto, ON M1C 1A4, Canada; lauren.hong@mail.utoronto.ca; 2 KITE Research Institute, Toronto Rehabilitation Institute, University Health Network, Toronto, ON M5G 2A2, Canada; 3 Department of Psychology, University of Toronto, Toronto, ON M5S 3G3, Canada; 4 Department of Language Studies, University of Toronto Scarborough, Toronto, ON M1C 1A4, Canada; chao.han@utoronto.ca; 5 Department of Linguistics, University of Toronto, Toronto, ON M5S 3G3, Canada

**Keywords:** mismatch negativity (MMN), accent processing, second language (L2), talker identity, speech perception, electroencephalography (EEG)

## Abstract

**Background:** Accented speech contains talker-indexical cues that listeners can use to infer social group membership, yet it remains unclear how the auditory system categorizes accent variability and how this process depends on language experience. **Methods:** The current study used EEG and the MMN oddball paradigm to test pre-attentive neural sensitivity to accent changes of English words *stopped* produced by Canadian English or Mandarin Chinese-accented English talkers. Three participant groups were tested: Native English listeners, L1-Mandarin listeners, and Heritage Mandarin listeners. **Results:** In the Native English and L1-Mandarin groups, we observed MMNs to the Canadian accented English deviant, indicating that the brain can group speech by accent despite substantive inter-talker variation and that this grouping is consistent with an experience-dependent sensitivity to accent. Exposure to Mandarin Chinese-accented English modulated MMN magnitude. Time-frequency analyses suggested that α and low-β power during accent encoding varied with language background, with Native English listeners showing stronger activity when presented with Mandarin Chinese-accented English. Finally, the neurophysiological response in the Heritage Mandarin group reflected a broader phonological space encompassing both Canadian English and Mandarin-accented English, and its magnitude was predicted by Chinese proficiency. **Conclusions:** These findings provide brain-based evidence that automatic accent categorization is not uniform across listeners but interacts with native phonology and second-language experience.

## 1. Introduction

Speech transmits both the linguistic message and talker-indexical characteristics [1]. From the acoustic signal alone—often with sparse exposure to a given talker—listeners reliably assess talker age [2,3,4,5], sex and gender [6,7,8,9], race and ethnicity [10,11,12,13], as well as physical attributes, such as height and weight [14,15]. Listeners also identify non-native accents with as little acoustic information as a single segment [16,17]. Such cues to talker accent and dialect are used to socially categorize individuals or establish group membership [18,19]. The current study examines whether the brain categorizes speech based on talker accent and whether language experience affects this categorization.

Spatial activity patterns in the superior temporal cortex highlight a functional dissociation between linguistic content and talker-indexical cues [20,21]. Phonetic information is encoded in overlapping superior temporal regions, including Heschl’s gyrus, the planum temporale (PT), the superior temporal gyrus (STG), the superior temporal sulcus (STS), and the middle temporal gyrus [22,23,24,25,26,27,28]. Conversely, repeated talker exposure drives right anterior STS activation patterns [29], although some spatial overlap is observed between speech recognition regions in left STS/STG and talker-specific vocal tract parameters [30]. Functional imaging also reveals enhanced activation of auditory and memory-related networks for familiar compared with unfamiliar voices, leading to improved performance in listening and word memory tasks [31,32]; using electrophysiology, familiar voices elicit larger amplitude event-related potentials (ERP) compared to unfamiliar talkers [33,34,35], and physical talker characteristics are encoded in the dynamics of early electrophysiological responses, while social characteristics emerge later [36]. Moreover, voice familiarity differences are observed in α-band desynchronization [37], while θ-band and γ-band activity dominate linguistic encoding [38,39]. Together, this evidence indicates at least partially distinct mechanisms for the neural processing of talker-indexical and linguistic cues.

Studies of forensic talker profiling indicate that non-native accent and regional dialect are identifiable features of an unknown voice and have been used in legal proceedings [40]. In instances where dialect and accent are probabilistically linked with marginalized groups, profiling leads to discrimination [41,42], the denial of access to housing [19,43,44], and triggers exclusionary behavior [45]. Linguistic profiling persists even in putatively socially conscious, younger individuals [46]. Despite these social and economic implications, the neurophysiological mechanisms for the grouping and categorization of individuals based on accent and dialect remain underexplored.

A powerful tool to investigate the neurophysiological categorization and grouping of speech is the mismatch negativity (MMN), an automatic and pre-attentive change detection ERP [47,48,49]. The MMN localizes to the supratemporal auditory cortex [48,50,51,52,53], making it suitable for testing talker-based categorization. Oddball paradigms, which present frequent standard stimuli interspersed with infrequent deviants, are commonly used to elicit the MMN. Detection of a deviant along perceptual or representational dimensions evokes a negative deflection in the ERP, typically peaking at 150–350 ms after stimulus onset and largest over fronto-central electrode sites. While traditional oddball paradigms involved the repeated presentation of physically identical standard stimuli [54,55,56], the MMN is also observed when within-category physical variation is introduced into the standard stimuli [57,58,59,60,61,62,63,64]. Relevant to the current design, changes in voice are sufficient to elicit an MMN [33,65], and the linguistic background of participants also impacts MMN response characteristics [66,67,68,69,70,71,72,73,74,75].

MMN latency is influenced by accent familiarity. An earlier MMN was observed when Standard American English listeners were exposed to acoustically variable African American English tokens of *hello* as standards and a Standard American English *hello* as the deviant; conversely, a later MMN was observed when the standard was in Standard American English and the deviant was in African American English [76]. The presence of MMNs in both configurations indicated that accent variation is categorized during early cortical phonetic processing, while latency differences reflect the influence of accent familiarity on the temporal and spatial dynamics of the MMN. Specifically, deviant stimuli produced in a familiar accent elicited an earlier MMN relative to deviants produced in an unfamiliar accent, suggesting that the familiar accent was psycho-acoustically more salient. In contrast, deviants produced in the less familiar accent elicited larger MMN responses in Standard German and Swiss-German participants, which was interpreted as the unfamiliar accent imposing greater processing costs [67]. That said, the MMN latency reported in Bühler et al. [67] was consistent with the MMN latency to the unfamiliar deviant reported in Scharinger et al. [76]. Furthermore, changes in talker-sex and talker-accent in an oddball paradigm result in the elicitation of the MMN response in native and non-native participants alike [77]; that said, the stimuli consisted of isolated vowels, with only one token per category, making it unclear whether listeners perceived standard-deviant differences as meaningful accent variation or merely low-level acoustic contrasts, especially as native and non-native listeners showed similar MMN responses. Overall, the extent to which language experience, particularly accent familiarity and phonological background, shapes neurophysiological processing of accent variability remains an open question.

Accent categorization requires grouping speech despite low-level, inter-talker acoustic variation. Previous MMN tests assessing accent processing have either used a single stimulus [33,67,77] or multiple stimuli produced by the same talker [76]. While this approach isolates accent information from other talker-specific cues, it leaves unaddressed the question of whether the brain generalizes across talkers to extract accent. The MMN has been employed to show that the brain posits generalizations on the basis of abstract phonological features despite substantial inter-category phonetic variation [57,60,62] and on the basis of vowel category despite substantial inter-talker variation [64]. The current study introduces inter-talker variability in the oddball paradigm to determine whether the auditory system can generalize an accent beyond individual talkers and the associated low-level acoustic differences.

Using an inter-talker varying oddball paradigm, the current study tested whether the brain automatically categorizes speech based on accent across individual talkers and whether the magnitude or timing of the brain response—as indexed by the MMN—is modulated by language experience. Following Scharinger et al. [76], we used whole-word English stimuli. In an oddball paradigm, we presented native English listeners, heritage Mandarin listeners, and L1-Mandarin learners of English with the word *stopped* produced in two accents (i.e., Canadian-accented English (CAE), Mandarin-accented English (MAE)) while their electroencephalogram (EEG) was recorded. Crucially, standard and deviant tokens were sampled from ten different talkers of each accent, requiring listeners to group talkers based on accent and ignore low-level acoustic variation due to changes between talkers. Overall, an accent change should elicit an MMN response if the brain perceptually groups multiple talkers from the same accent together. Both Scharinger et al. [76] and Bühler et al. [67] report an accent-change-induced MMN. That said, the reported latencies and amplitudes differed depending on deviant familiarity. Consistent with previous studies that report an earlier and larger MMN to familiar and more frequent deviants [33,34,35,76,78], we predict a larger and earlier MMN to the familiar accent. Specifically, we predict a larger MMN to the CAE deviant in native English listeners and a larger MMN in the L1 Mandarin participants to the MAE deviant. Given their language background, heritage Mandarin listeners were expected to be familiar with both Standard Canadian English and Mandarin-accented English, thus exhibiting greater tolerance to accent variability and, potentially, an overall reduced MMN. Finally, in the oscillatory dynamics of the electrophysiological responses [37], we predict differences in the familiar accent to result in larger α-band desynchronization.

## 2. Methods

### 2.1. Participants

Sixty-nine participants (47 females; mean age: 19.1 years, range: 17–24 years) were recruited from the University of Toronto community and received course credit for participation. All participants self-reported no known hearing, language, neurological, or visual deficits. Prior to the experiment, participants provided written informed consent and completed a language background questionnaire, which queried age of acquisition, self-rated language proficiency scores (10-point Likert scale; 1: least proficient, 10: most proficient), daily use percentage for each reported language, and familiarity with and likelihood of exposure to Mandarin-accented English (10-point Likert scale; 1: minimal familiarity/exposure; 10: high familiarity/exposure). Accent familiarity and exposure ratings were included in analyses to examine the impact of language experience on the neurophysiological responses. Four participants were excluded due to technical errors during recording. The remaining 65 participants were categorized into the following groups: Native English listeners (*n* = 22) with no formal or informal experience learning Mandarin Chinese; Heritage Mandarin listeners (*n* = 17), who were raised in an English-speaking community, but whose parent(s) spoke Mandarin; and advanced L1-Mandarin learners of English (*n* = 26), who received formal English instruction and were enrolled at the University of Toronto. Table 1 provides participant demographics. As expected, Native English listeners reported the lowest familiarity with MAE, whereas Heritage Mandarin listeners and L1-Mandarin listeners showed comparable MAE familiarity. For likelihood of exposure, English listeners also reported the lowest rating, while L1-Mandarin listeners reported the highest rating. The experiment was approved by the Research Ethics Board of the University of Toronto.

### 2.2. Materials

Experimental stimuli were obtained from the recordings by 10 native Canadian-accented English (CAE) talkers and 10 L1 Mandarin Chinese-accented English (MAE) talkers. L1 Mandarin talkers were all born in China (mean length of residence in Canada: 1.83 years, range: 5 months–2 years). CAE talkers were of Chinese descent but were either born in Canada or arrived in Canada before 5 years of age [41,42]. Acoustic recordings were made with a Røde NT5 condenser microphone (Sydney, Australia) in a sound-attenuated cabin. Sound files were recorded at a 44.1 kHz sampling rate and 16-bit depth encoding on a Mix-Pre 3 (Sound Devices, Reedsburg, WI, USA) soundcard. Talkers read a list of seventeen words, repeated six times. Words were selected to contain phonetic properties (e.g., syllable structures, phonemes) that are commonly difficult for L1 Mandarin Chinese English learners to produce and had been previously reported to be familiar to L2 East Asian English speakers [79]. All items were monosyllabic real words of English, included a complex coda, e.g., [p^h^ɑkt] “packed”, [ɡlɪmps] “glimpse” [80], and contained one of the following vowels: [æ ʌ ai] [81].

The English word *stopped* was selected as the test item, as the authors deemed it to have the most consistent pronunciation across talkers of the same accent from the word list. Only recordings from female talkers that were clear, produced at a comfortable speech rate, and did not deviate from the common pronunciation among this group were included (*n* = 36). Then, the most tonally neutral repetition per talker was extracted as assessed by the first author. All stimuli were digitally scaled to have an equal root mean square (RMS) intensity in *Praat* [Version 6.5.54] [82]. To identify the test items, a seven-point auditory Likert rating task (1 = little accent relative to CAE; 7 = very accented relative to CAE) was administered online to naïve, native Standard Canadian English participants (*n* = 17). One token of the word *stopped* for each talker was presented in pseudorandom order. Participants completed the accentedness rating task on an online form. The ten CAE talkers that were judged to be least accented relative to CAE (median = 1, *SD* = 0) and the ten L1 Mandarin talkers that were judged to be most accented (median = 6; *SD* = 0.95) were selected for inclusion in the main experiment. Inter-rater reliability, assessed by an intra-class correlation coefficient (two-way random-effects model) [83], was excellent (ICC2k = 0.97, 95% CI [0.95, 0.99], *p* < 0.001). Acoustic measurements for the experimental stimuli are provided in Table 2. The two accents most reliably differ in the duration of initial [s] and in the closure duration of [t] in the [st] cluster [84]. See Supplementary Materials for talker ratings and visualizations of the acoustic variables. Individuals who recorded experimental items were excluded from participation in the EEG experiment.

### 2.3. Procedure

Participants were seated in a quiet, dimly-lit room and passively listened to the stimuli, while watching a silent movie to maintain an awake state and reduce excessive ocular movements [85]. An auditory oddball paradigm was used to elicit the MMN. Auditory stimuli were delivered via Beyerdynamic DT 770 PRO headphones (Heilbronn, Germany) calibrated for 70 dB SPL auditory playback; the sound presentation level was constant across participants. The experiment consisted of two blocks. In the MAE-deviant block, participants were presented with the English word *stopped* produced by CAE talkers as the standard. The deviant was also the English word *stopped* but produced by MAE talkers. In the CAE-deviant block, participants were presented with the English word *stopped* produced by MAE talkers as the standard. The deviant was also the English word *stopped* but produced by CAE talkers. In both blocks, the experimental tokens were sampled from the twenty different female talkers selected for use in the EEG experiment, ten from each accent group. We opted to present multiple talkers for each accent group, as the selection of just one talker per accent group would have confounded accent change with voice change, and voice changes have been known to elicit MMN responses [65]. The current study presented multiple talkers to ensure that neurophysiological responses reflected abstract categorizations based on accent rather than individual acoustic properties [61,86]. Block order was randomized across participants.

Each deviant was preceded by approximately eight standard stimuli (range: 4–10) for the elicitation of an MMN response [68,87]. The number of standards preceding each deviant was randomly drawn from a uniform distribution. Seventy deviants were presented in each block. Individual tokens were randomly sampled from the ten tokens that belonged to the standard accent or from the ten tokens that belonged to the deviant accent. The interstimulus interval duration was randomly sampled from a uniform distribution between 0.7 s and 0.95 s. The experimental design was a mixed 3 (group: Native English Listeners, Heritage Mandarin Listeners, L1-Mandarin Learners) × 2 (block: CAE-deviant, MAE-deviant) × 2 (stimulus: standard, deviant) design, with group being a between-participants factor and both block and stimulus being within-participants factors.

### 2.4. EEG Recording and Analysis

Continuous EEG recordings were acquired from 32 actiCAP active electrodes connected to an actiCHamp amplifier (Brain Products GmbH, Gilching, Germany). The EEG signal was digitized at a 500 Hz sampling frequency with a 200 Hz on-line low-pass filter. Electrodes were positioned on the scalp according to the International 10–20 system. Positions included Fp1/2, F3/4, F7/8, FC1/2, FC5/6, FT9/10, C3/4, T7/8, CP1/2, CP5/6, TP9/10, P3/4, P7/8, O1/2, Oz, Fz, Cz, and Pz. A ground electrode was placed at Fpz. The EEG signal was referenced to the right mastoid (TP10) on-line. Impedances were reduced to below 10 kΩ at each electrode site prior to recording. The experiment was deployed using PsychoPy [Version 3.0.0b10] [88]. In addition to the EEG channels, the auditory signal was also sent to the amplifier using the StimTrak device (Brain Products GmbH, Gilching, Germany). This allowed for off-line correction of temporal delays between the delivery of the auditory stimulus and the digital trigger marker [89].

EEG recordings were preprocessed using MNE-Python [Version 1.6.1] [90]. A band-pass filter from 1 to 100 Hz was applied to the continuous EEG signal, which was then downsampled to 250 Hz. Bad channels were identified and interpolated using the PyPREP toolbox [91]. Independent Component Analysis (ICA) was performed to identify and remove artifacts. To run the ICA, data were average re-referenced and segmented into epochs ranging from −100 to 900 ms relative to stimulus onset. ICA was performed using the extended infomax algorithm, and components were automatically classified using the ICLabel classifier [92]. Components not classified as *brain* or *other* within the first 15 components (ranked by variance explained) were excluded. All classifications were manually inspected to ensure that salient artifacts (e.g., eye blinks, saccades) were rejected (mean = 6 components per participant, SD = 2). The ICA solution was then applied to the continuous data, which were subsequently re-referenced to the average linked mastoid [93].

### 2.5. EEG Analysis

#### 2.5.1. Event-Related Potentials

The preprocessed continuous EEG signal was segmented into epochs time-locked to stimulus onset, spanning −100 ms pre-stimulus to 900 ms post-stimulus, chosen as the longest stimulus duration was 830 ms. Epochs with peak-to-peak amplitudes greater than 100 µV were rejected (accounting for 3% of total trials). To ensure that the ERP to standard tokens reflected an established memory trace of the abstract accentual category, the first two standards in each standard train were excluded. For each participant, standards and deviants were averaged separately in each block. The identity MMN (iMMN) was computed for each accent by subtracting the ERP to the accent serving as standards in one block from the ERP to the same accent serving as deviants in the other block [94]. This was done to isolate the MMN response from ERP differences intrinsic to physical property differences between stimuli. For example, the CAE iMMN was calculated by subtracting the ERP to CAE standards in the MAE deviant block from the ERP to CAE deviants in the CAE deviant block. The presence of an iMMN suggests that observed differences are attributable to accent status rather than physical stimulus properties. Inferential statistics were conducted using the spatiotemporal cluster-based permutation test applied to the 0–900 ms time interval. A two-tailed *t*-statistic threshold corresponding to *p* = 0.05 identified time samples and electrodes showing significant deviation from zero. Cluster-based correction for multiple comparisons was applied with a *p* = 0.05 significance threshold. For each significant cluster, we report cluster mass, defined as the sum of *t*-values over samples within the cluster, cluster *p*-value, and the cluster extent quantified by the number of time points and electrodes. Furthermore, deviant–standard difference waves were averaged across all cluster samples for each participant, and Cohen’s *dz*—the mean of participant-wise cluster averages divided by their standard deviation—was quantified as the effect size [95].

#### 2.5.2. Event-Related Spectral Perturbation

To further explore the mechanisms underlying accent processing, we computed event-related spectral perturbations (ERSP) to determine how accent modulates the oscillatory dynamics in each participant group (Native English listeners, Heritage Mandarin listeners, L1-Mandarin listeners). The ERSP epochs were extracted from the preprocessed continuous EEG signal using a time window from −1.0 s to 2.0 s relative to stimulus onset. Artifact rejection was applied using a peak-to-peak threshold of 100 µV (7% of trials). The time-frequency decomposition was performed using a Morlet wavelet convolution. Frequencies ranged from 3 to 30 Hz, linearly spaced across 28 steps. The number of wavelet cycles increased linearly with frequency, starting at 3 cycles and increasing by 0.8 steps per frequency bin. This approach balances temporal and spectral resolution. ERSPs were computed for each trial and averaged across epochs and all EEG channels for each standard and deviant condition in each group. Power estimates were baseline corrected using log-ratio normalization, with a −450 to −300 ms pre-stimulus baseline time window. This time window permits examination of pre-stimulus predictive coding [60,96], as it minimizes the contamination of baseline power estimates in the pre-stimulus predictability window, given temporal smearing in time–frequency decomposition. Group-level statistical comparisons were conducted using a cluster-based permutation test [97], with cluster adjacency defined across spatial (channel), spectral (frequencies), and temporal (time samples) dimensions. The threshold for initial cluster formation was set to the *t*-value corresponding to *p* = 0.01 (two-tailed), and statistical significance was assessed using a cluster-level permutation at *p* < 0.05. Analyses were restricted to the 3–30 Hz frequency range and the −200 to 900 ms post-stimulus interval.

#### 2.5.3. Generalized Additive Modeling

To assess whether brain responses to MAE are modulated by individual experience with Mandarin, we implemented generalized additive models (GAMs), which have been reported to be more reliable predictors of language background than mean-amplitude measures [98]. GAMs model ERP dynamics as a non-linear function of time, leveraging the entire time series. This approach reduces reliance on manually selected time windows in traditional mean-amplitude analyses and better reflects individual differences in waveform shape and component latency, which may arise from nuanced language experience variation. We built GAMs using the bam() function from the *mgcv* package [99] in *R* [100] to capture non-linear temporal dynamics in EEG signals, resulting in the following model:

μV ~ s(Time, by = Condition) + s(Time, Item, bs = “fs”, m = 1)

*Condition* represents the Block (CAE-deviant, MAE-deviant) × Stimulus (standard, deviant) interaction. *Item* refers to the specific auditory stimulus. The term *s(Time, Item, bs = “fs”, m = 1)* allows for a separate smooth over time for each stimulus, accounting for item-level variability and penalizing overfitting. The model was applied to single-trial EEG data, averaged over eight frontocentral electrodes (i.e., Cz, Fz, FC1, FC2, CP1, CP2, C3, C4), where the MMN response is observed [53]. For each participant and each accent, a difference ERP (i.e., deviant ERP minus standard ERP, for the same accent across blocks) was derived from the model-fitted values using the difference_smooth() function from the *gratia* package [101]. Based on the model-derived difference waveform, the MMN component was identified as the negative deflection containing the global negative peak within the 0–900 ms post-stimulus interval. For each participant and each accent condition, two individual-level measures were extracted. First, the normalized modeled peak was calculated. This is the peak amplitude of the identified MMN component, divided by 1.96 times the standard error of the fitted value at that time point. This measure indicates response robustness and has been reported to reliably reflect language background [98]. Second, the half-area latency was calculated. This is the time point at which 50% of the total area under the negative deflection has been reached, representing the temporal dynamics of the brain response [102]. The latter measure was motivated by the findings reported in Scharinger et al. [76], who observed MMN timing differences due to dialect familiarity.

In addition to the GAM-derived measures, we also extracted a traditional ERP difference measure for each participant, defined as the mean deviant-minus-standard amplitude within the time window and electrode region selected based on visual inspection of the grand-average different waveform and its scalp distribution. These three individual-level neural measures were then correlated with participant responses regarding experience with MAE, including the familiarity and the likelihood of exposure, as well as their Chinese language proficiency (for Heritage Mandarin listeners only).

## 3. Results

### 3.1. Event Related Potentials

**Figure 1:**
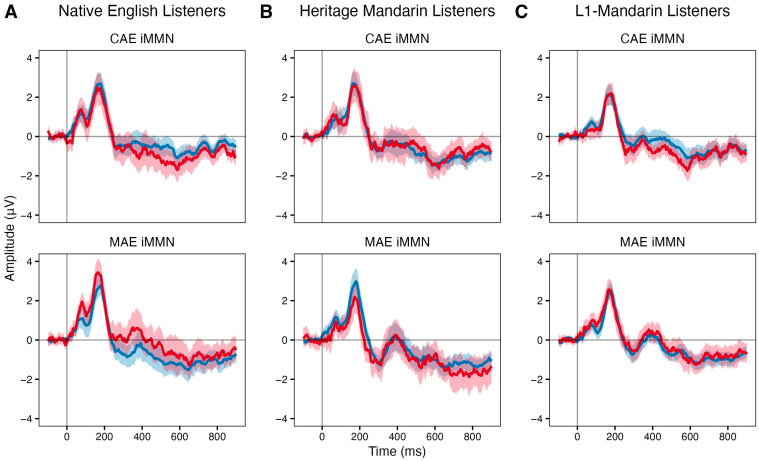
Event-related potentials (ERP) to standards (blue) and deviants (red) averaged over frontocentral electrodes for Native English listeners (**A**), Heritage Mandarin listeners (**B**), and L1-Mandarin listeners (**C**). For each panel, the top row shows ERPs to CAE (Canadian-accented English) as the standard (from the MAE-deviant block) and deviant (from the CAE-deviant block); the bottom row shows ERPs to MAE (Mandarin Chinese-accented English) as the standard (from the CAE-deviant block) and deviant (from the MAE-deviant block). Shaded regions represent the 95% confidence interval of the mean ERP waveform.

Figure 1 displays the ERP waveforms for each group, averaged over the eight frontocentral electrodes (Fz, FC1, FC2, Cz, C3, C4, CP1, CP2). For CAE (upper panel), a clear negative deflection for CAE deviants relative to CAE standards was observed in both Native English listeners (Figure 1A) and L1-Mandarin listeners (Figure 1C).

**Figure 2:**
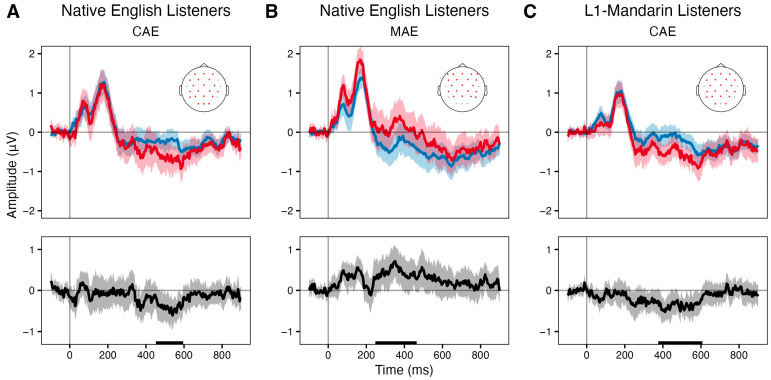
Event-related potentials (ERP) to the standard (blue) and deviant (red) stimuli, and corresponding difference waveforms (deviant—standard, black) averaged over electrodes within the significant cluster (highlighted in red in the topography). (**A**) ERPs to CAE as standard (from the MAE-deviant block) and deviant (from the CAE-deviant block) in Native English listeners. (**B**) ERPs to MAE serving as the standard (from the CAE-deviant block) and deviant (from the MAE-deviant block) in Native English listeners. (**C**) ERPs to CAE as standard (from the MAE-deviant block) and deviant (from the CAE-deviant block) in L1-Mandarin learners. Shaded areas represent the 95% confidence interval of the ERP waveform. Rug plots along the x-axes mark time samples within the significant cluster. Only conditions associated with significant clusters are shown.

**Figure 3:**
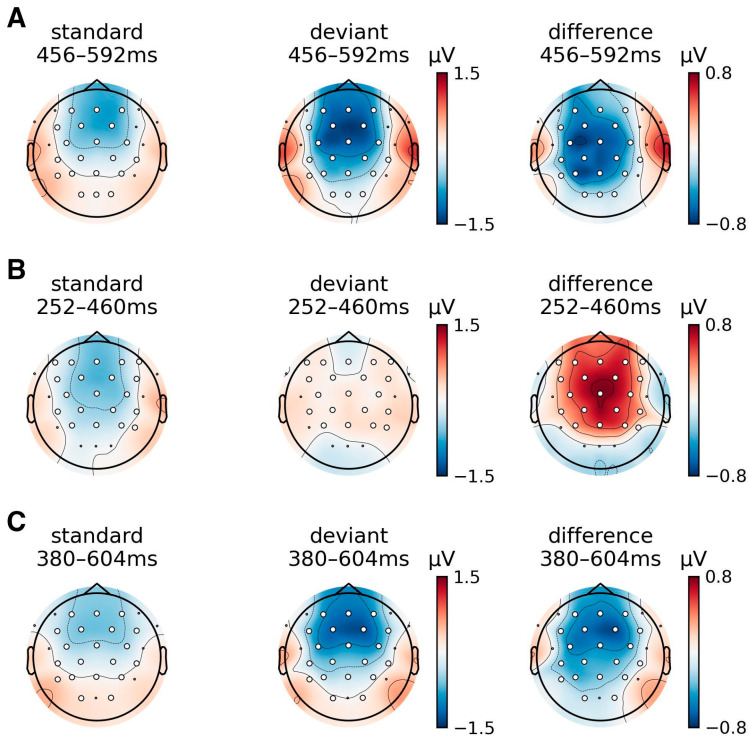
Scalp ERP topographies averaged over time samples identified by the spatiotemporal permutation test for the standard (**left**), deviant (**middle**), and their difference (deviant—standard, **right**). (**A**) CAE in Native English listeners. (**B**) MAE in Native English listeners. (**C**) CAE in L1-Mandarin listeners. Only conditions associated with significant clusters are shown. Electrode sites marked with white circles indicate where there was a significant difference identified within the time window.

To determine whether accent deviation elicited a robust iMMN response regardless of talker variability, we conducted cluster-based permutation tests on the difference ERP waveforms, derived from comparing each accent when presented as deviants against the same accent when presented as standards. This approach offers a direct comparison to Scharinger et al. [76], who also computed the iMMN. Figure 2 and Figure 3 display the waveforms and topographies, respectively, corresponding to the permutation test results. Waveforms were averaged over the electrodes contributing to the significant spatiotemporal clusters, and the topographies reflect ERP activity averaged over significant time windows. For Native English listeners, two significant clusters were identified: The first cluster (cluster mass = −760.05, cluster *p* = 0.029, *dz* = −0.67) spanned 456–592 ms (35 time points) and included 20 electrodes. This cluster reflected the difference between the CAE deviant (from the CAE-deviant block) and the CAE standard (from the MAE-deviant block). The waveform revealed that the CAE deviant elicited a more negative response, and the frontocentral distribution of this difference aligns with classic MMN topographies [48]. The second cluster (cluster mass = 1040.23, *p* = 0.027, *dz* = 0.59) spanned (53 time samples) and included 20 electrodes, showing a frontocentral positivity for the MAE deviants compared to the MAE standards. The reversed polarity is not typical of MMN responses but aligns with previous oddball studies when the standard is the unmarked category [57,60]. For L1-Mandarin listeners, one cluster was found (cluster mass = −1332.50, *p* = 0.0103, *dz* = −0.67), spanning 380–604 ms (57 time samples) across 20 electrodes. The topography showed a frontocentral negativity for the CAE deviant relative to the CAE standard, mirroring the pattern in Native English listeners and supporting the presence of a late MMN. No clusters were observed for the MAE deviant. In contrast, Heritage Mandarin listeners showed no clusters for either accent contrast, suggesting an attenuated iMMN response. To quantify the null effect, we computed effect sizes and conducted a sensitivity analysis based on mean deviant–standard amplitude differences in a late window (300–600 ms) averaged over the eight electrodes. The time window was chosen based on inspection of the grand-average difference waves across all three participant groups. The mean difference in the Heritage Mandarin group was near zero (mean = 0.12 µV, *dz* = 0.15); with the current sample size (*n* = 17), the experiment had 80% power (*α* = 0.05, two-tailed) to detect effects of Cohen’s *dz* over 0.72. While smaller effects cannot be ruled out, the absence of a significant cluster in the Heritage Mandarin group does not support an MMN effect of a moderate effect size, as observed in Native English and L1-Mandarin groups, and is unlikely to be explained solely by the smaller sample size.

To summarize, iMMN responses to CAE were observed in both Native English listeners and L1-Mandarin learners of English, suggesting that in both populations, the auditory cortex robustly detected accent-based deviations. In contrast, this accent-based neural discrimination was absent in Heritage Mandarin listeners, suggesting that these participants may have treated CAE and MAE as belonging to the same category or as insufficiently distinct to trigger an automatic deviance response.

### 3.2. Event-Related Spectral Perturbation

**Figure 4:**
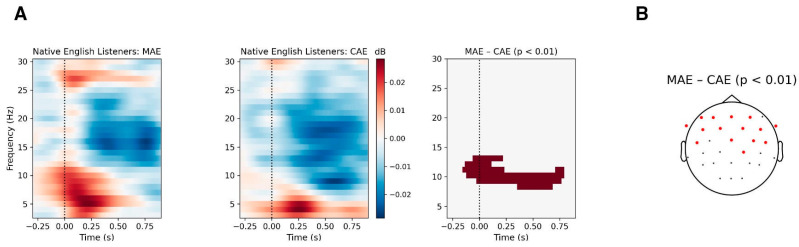
Cluster-based permutation test comparing the CAE standard and the MAE standard in Native English listeners. (**A**) ERSP for CAE standards (**left**) and MAE standards (**center**). The (**right**) panel shows the time-frequency bins for the significant cluster. (**B**) Topographic distribution of electrodes in the significant cluster at one or more time-frequency bins marked in red. Dashed lines indicate stimulus onset.

To assess whether neural activity underlying the accent difference detection varied across groups, we analyzed the oscillatory power of the EEG responses to the standard tokens. Given the lack of a specific hypothesis regarding relevant frequency bands, we conducted a time-frequency analysis spanning 3–30 Hz and extending to 900 ms post-stimulus onset. Permutation tests comparing the two standard ERSPs in Native English listeners revealed a significant cluster (cluster mass = 3118.56, cluster *p* = 0.005, *dz* = 1.31) spanning 8–13 Hz and −160 to 788 ms (80 time samples) across 15 electrodes in which the MAE standard elicited increased oscillatory power relative to the CAE standard (Figure 4). Visual inspection of the cluster suggested increased power in the low-β band (12–13 Hz) approximately −150 ms pre-stimulus to 250 ms post-stimulus and in the α-band (8–12 Hz) sustaining up to 750 ms post-stimulus. The increase in low β-power aligns with prior work suggesting that β-activity reflects top-down predictive coding [60,103], potentially indicating that the MAE standard was perceived as less expected. The sustained enhancement in α-power likely reflects greater processing difficulty [104] or top-down inhibition [105] when encoding the less familiar MAE speech. In contrast, no ERSP differences between CAE and MAE standards were observed for either Heritage Mandarin listeners or L1-Mandarin listeners, intimating comparable oscillatory dynamics for the two accent types in bilingual listeners. This may reflect increased experience with both accents and hence a reduced neural distinction between them at the level of predictive and attention-related oscillatory activity.

### 3.3. Generalized Additive Models

To examine how neurophysiological responses were modulated by individual language experience and whether such relationships differed across language groups, we extracted individual-level brain measures from fitted GAM models. These measures included normalized modeled peak and half-area latency, which index the magnitude and timing of the MMN response, respectively. For comparison with previous studies, we also computed a traditional amplitude measure of the MMN, defined as the mean amplitude difference averaged over the 400–600 ms time window and eight frontocentral electrodes (i.e., Fz, FC1, FC2, CP1, CP2, Cz, C3, C4). For each language experience variable (i.e., MAE familiarity, MAE likelihood) and for each MMN condition (i.e., iMMN to MAE deviant, iMMN to CAE deviant), we fit linear regression models regressing the MMN measure on group (Native English listeners, Heritage Mandarin listeners, L1-Mandarin listeners), rating, and their interaction. Interactions were followed up using the *emmeans* package [106] to estimate slopes within each group and to test whether the slopes differed across groups.

**Figure 5:**
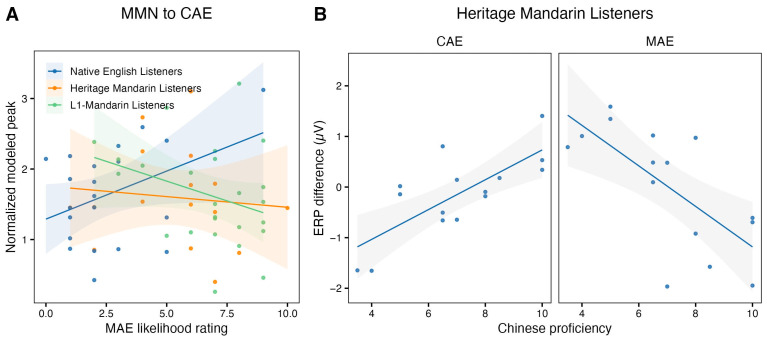
(**A**) MAE likelihood ratings by the normalized modeled peak (positive values indicate stronger MMNs) for each language group. (**B**) Self-reported Chinese proficiency by the traditional amplitude MMN measure (negative values indicate stronger MMNs) for Heritage Mandarin listeners. Shaded regions represent the 95% confidence interval of the mean.

For the normalized modeled peak measure, the model revealed a marginal group × MAE-likelihood rating interaction: *F*(2, 56) = 3.08, *p* = 0.05. Within-group slopes did not reach significance (all *p*s > 0.05). Nonetheless, follow-up pairwise slope comparisons suggested opposite directions across groups, *t*(56) = 2.46, *p* = 0.04: Native English listeners exhibited a positive correlation where greater likelihood of hearing MAE was associated with a larger MMN response to the CAE deviant (Figure 5A); In contrast, L1-Mandarin listeners showed a negative slope: greater exposure to MAE correlated with reduced MMN responses to the CAE deviants. No correlations were observed for half-area latency or for the traditional amplitude measure.

For Heritage Mandarin listeners, we examined whether the MMN was predicted by Chinese proficiency by correlating each MMN measure (i.e., normalized modeled peak, half-area latency, traditional amplitude measure) with participants’ self-rated proficiency scores. Since Chinese speaking and listening ratings were correlated (*r* = 0.57, *p* < 0.001), we averaged the two to derive a single composite proficiency score. Only the traditional ERP difference measure showed an association with proficiency (Figure 5B): higher Chinese proficiency predicted more positive deviant–standard difference responses to the CAE deviant (*r* = 0.75, *p* = 0.001) but was also associated with more negative difference responses to the MAE deviant (*r* = −0.7, *p* = 0.004).

## 4. Discussion

The present study tested whether the auditory system pre-attentively generalizes accent beyond inter-talker variability, and whether such neurophysiological categorization is shaped by language experience. Using a multi-talker oddball design, we observed an MMN in Native English listeners and L1-Mandarin learners to CAE deviants, indicating that the brain can group speech by accent and that accent change can be detected despite talker-specific variation. MMN responses emerged relatively late, approximately 400–600 ms post-stimulus, consistent with prior reports of late MMN responses to complex speech stimuli [107,108] and with evidence that abstract social information derived from voices emerges later in the electrophysiological response [36]. As such, the current MMNs reflect the integration of information over the unfolding word and extraction of higher-order regularities relevant to accent perception across multiple talkers.

For Native English speakers, we predicted a larger MMN to the familiar CAE deviant than to the unfamiliar MAE deviant. We observed a frontocentral MMN to the CAE deviant, whereas the MAE deviant elicited a frontocentral positivity. We interpret these differences in polarity as reflecting distinct neurophysiological processing mechanisms. The MMN to the CAE deviant might reflect a native-accent advantage, consistent with prior results reporting an earlier and enhanced MMN to a familiar accent [76]. Processing the familiar accent may be supported by a more stable long-term representation, increasing neural sensitivity to the familiar deviant occurring in a less familiar (i.e., MAE standards) context [35]. This interpretation aligns with broader evidence that familiarity can sharpen auditory representations, leading to larger N1 and P2 responses to familiar versus unfamiliar non-linguistic sounds [109], larger MMNs for higher-frequency word deviants [78], and larger MMNs to deviant voices of familiar individuals relative to unfamiliar talkers [33].

At first glance, this pattern appears to contrast with Bühler et al. [67], which reported a reduced MMN when more familiar vowel allophones served as deviants in the context of less familiar allophones. They interpret this as greater processing efficiency for familiar allophones and hence less prediction error. Importantly, however, Bühler et al. [67] also emphasized that familiarity effects can differ between within-category allophonic contrasts and higher-level contrasts, and that the amplitude modulation of the familiarity-driven MMN is not necessarily invariant across processing domains and stimulus structures. In the present study, the stimuli were words, and the extracted regularity was accent patterns across talkers. Thus, the effect of familiarity may primarily manifest as enhanced sensitivity to the familiar accent rather than reduced prediction error. By contrast, the MAE deviant yielded a more positive-going response over frontocentral sites in an earlier time window (200–400 ms). We interpret this positivity as driven by a P3a response for the MAE deviant occurring among CAE standards. The P3a has been linked to the processing of complex deviants and automatic orienting responses to novelty [35,110,111]. Accordingly, the MAE deviant may be more salient and elicit stronger involuntary orienting as it was less familiar to the Native English listeners. Time-frequency results provide converging evidence that MAE imposed greater processing demands: MAE standards elicited increased α (8–12 Hz) and low-β (12–13 Hz) power relative to the CAE standards in Native English listeners. Oscillatory β-band responses have been linked to top-down signaling and prediction maintenance in speech perception [96,105]. Notably, the effect emerged in the pre-stimulus interval (−150 to 250 ms). The timing is consistent with prior work reporting pre-stimulus β-band increases, interpreted as anticipatory activity associated with maintaining a top-down predictive state [60,96]. We interpret the pre-stimulus low-β power increase to the MAE standards as reflecting stronger maintenance of top-down predictions of the upcoming input in Native English listeners when processing an unfamiliar accent. In parallel, enhanced α-power indexes greater cognitive demand and effortful suppression of competing information in challenging auditory processing contexts [104,105]. Increased α-band desynchronization has been reported for familiar speech [37]. For Native English listeners, MAE may have led to greater processing difficulty and lower intelligibility, as it was less familiar, thereby increasing the need for sustained control (β-power) and effort/inhibitory regulation (α-power) during neurophysiological encoding of the standard.

In contrast, L1-Mandarin learners did not show a familiarity-driven MMN when MAE served as the deviant, despite the observation of an MMN to the CAE deviant. One explanation for this unexpected pattern, that is, a familiarity effect in Native English listeners but not in L1-Mandarin listeners, is that the observed MMN was driven by low-level auditory processing rather than accent-level categorization. Specifically, the same contrast tested in opposite directions (e.g., A deviants among B standards versus B deviants among A standards) can yield different mismatch responses. Non-linguistic directional MMN asymmetry has been observed for decrement versus increment in both the temporal domain [112,113] and the spectral domain [114]. Although the precise neural mechanisms remain unknown, such asymmetries may arise because increment and decrement changes recruit partially distinct change-detection processes, as equal physical changes are not necessarily perceptually symmetric. Under this view, if the CAE deviant was more acoustically salient when embedded among MAE standards than in the reverse configuration, then a larger MMN for the CAE deviant could emerge without requiring accent-level categorization; however, a purely acoustic account cannot explain why Heritage Mandarin listeners did not show a similar asymmetry (i.e., MMN to CAE but not to MAE) and showed no reliable MMN to either accent, albeit with the smaller sample size. Furthermore, a purely auditory account based on low-level acoustic contrast does not explain the group difference in oscillatory patterns, that is, the increased low-β/α power during standard encoding of MAE relative to CAE in Native English listeners but not in L1-Mandarin learners. This discrepancy suggests that the processes contributing to the MMN involve factors beyond acoustic change detection alone.

Considering the demographic profile of the L1-Mandarin participants, we speculate that the MMN to CAE deviants also reflects an experience-based familiarity advantage. Previous work on L2 speech perception suggests that as L2 experience and proficiency increase, neurophysiological indices of L2 speech processing tend to be more native-like [115,116,117]. The current L1-Mandarin listeners were advanced English users living in an English-speaking community, attended university classes taught in English, and routinely communicated in English with English-speaking peers. Therefore, even if they encountered MAE more often and might understand MAE more easily relative to Native English listeners, immersion in an English-speaking environment likely led to greater CAE familiarity than MAE, supporting robust CAE perception. While post-hoc, this interpretation is broadly compatible with evidence that L2 listeners show increased sensitivity to morphosyntactic violations when the L2 is produced in the target-language accent, relative to a non-native-accented version [118]. Since our stimuli were English words, and as such, were L2 lexical items for the L1-Mandarin participants, extensive L2 experience with English lexical items may place learners in a perceptual state that is more tuned for CAE relative to MAE, which could further contribute to the MMN to CAE. At the same time, given their high English proficiency, CAE may not impose sustained lexical processing demands relative to MAE, explaining the absence of a low-β/α power difference between MAE and CAE standards in L1-Mandarin learners. To summarize, familiarity may still contribute to the observation of an MMN to CAE in both Native English listeners and L1-Mandarin learners, but the additional processing challenges indexed by sustained low-β/α differences are specific to Native English listeners, rather than a uniform consequence of accent familiarity across groups.

Regarding Heritage Mandarin listeners, we found no spatiotemporal clusters for either accent deviant, and no ERSP difference between CAE and MAE standards. These null results suggest that, for Heritage Mandarin listeners who began acquiring both Mandarin and English early in life, MAE and CAE may be accommodated within an expanded phonological space. In other words, the deviant accent may be perceived as part of the expected realizations compatible with the accent representation of the standard accent and, therefore, does not trigger a pre-attentive change-detection response under high talker variability. This interpretation aligns with perceptual learning studies showing that exposure to accented speech supports adaptation, such that accent variability is treated as expected rather than salient [119,120]. This adaptation can involve down-weighting acoustic cues that deviate from expectations and learning accent-specific phonological regularities [121,122]. Together, these findings suggest that experience may promote broader grouping within a shared phonological space rather than distinct neural differentiation between accents. Overall, the MMN and ERSP results suggest that the auditory system can abstract an accent-level template that generalizes over inter-talker variability. Moreover, the MMN group differences are consistent with experience-dependent familiarity effects, aligning with prior findings of enhanced sensitivity to familiar voices [33,123], more efficient lexical access [124], and grammatical error detection in speech produced in a native accent [125,126].

GAM-based individual-difference analyses suggested that individual accent experience may reshape the accent perceptual space in a language-specific manner. Specifically, for Native English listeners, a higher likelihood of hearing MAE was associated with a larger MMN to CAE deviants, whereas for L1-Mandarin listeners, greater MAE exposure was associated with a reduced MMN to CAE deviants. It is possible that, for Native English listeners, increased contact with MAE sharpens the accent contrast such that MAE becomes a more clearly delimited accent category, yielding stronger predictions based on the MAE standards, hence a stronger prediction error elicited by CAE deviants. For L1-Mandarin listeners, increased MAE exposure may instead increase overlap between MAE and CAE within a broadened English phonological space, reducing sensitivity to CAE deviants. In this sense, exposure can either increase contrast (e.g., category sharpening) or increase tolerance (e.g., category blending), depending on the learner’s starting point and the functional role of MAE in daily input; however, this interpretation is tentative as the interaction was marginal and within-group slopes were not significant.

Within Heritage Mandarin listeners, Chinese proficiency modulates accent processing in a graded manner. At lower Chinese proficiency levels, the pattern resembles that of Native English listeners: CAE deviants elicited a more negative deviant–standard difference (i.e., more MMN-like), consistent with a familiarity advantage for CAE. In contrast, MAE deviants elicited a more positive-going deviant–standard difference response, consistent with a P3a-like orienting response to less familiar and therefore more salient deviants [35,110,111]. As Chinese proficiency increases, the responses shifted in a manner suggesting a broadened tolerance to accent variability. Specifically, the CAE deviant–standard difference became less negative (i.e., less MMN-like) as Chinese proficiency increased, whereas the MAE deviant–standard difference became less positive-going (i.e., less P3a-like) as Chinese proficiency decreased. Together, these shifts suggest a reduced familiarity-driven MMN for CAE deviants and a reduced novelty-driven orienting to MAE deviants, as MAE became less salient with increased Chinese proficiency. Higher Chinese proficiency may facilitate a broader phonological space in which MAE becomes more acceptable as an allophonic variant; however, it is unclear why the higher proficiency participants showed a more negative-going response for MAE than CAE. This trend requires further justification in future work. Overall, these patterns suggest that heritage experience places listeners along a continuum of accent representations that re-weight familiarity and salience.

We note two limitations. First, while we aimed for balanced group sizes, the Heritage Mandarin listeners group was the smallest of the three samples, which may have limited the statistical power required for detecting interactions. That said, the power analysis indicated that the sample size was approximately adequate to detect MMN differences with similar magnitudes as observed in the other two groups. Second, our stimuli were isolated English words presented in a passive MMN paradigm, which increases experimental control but limits generalization to accent processing in naturalistic contexts. In continuous speech, accent perception is also affected by syntactic and semantic information [127]; future work should therefore test whether the present effects replicate with higher-level linguistic structures, and evaluate generalizability across active tasks (e.g., accent judgment tasks), as well as across other accent pairings and listener populations (e.g., Mandarin monolingual participants and English learners of Mandarin perceiving English-accented Mandarin words).

## 5. Conclusions

The current study provides neurophysiological evidence that the auditory system can extract accent-level regularities that generalize across talker variability. This is reflected in the MMN to the CAE deviant in both Native English and L1-Mandarin listeners. Accent processing is also shaped by language experience: familiarity and language background influence accent differences both in deviant detection and in oscillatory processing dynamics. Specifically, Native English listeners showed a familiarity-driven MMN, as well as an ERSP difference, during standard processing, consistent with greater processing demands for MAE relative to CAE, whereas L1-Mandarin listeners showed an MMN to CAE deviants but no corresponding ERSP difference, consistent with more comparable processing efficiency across accents. Finally, heritage experience appears to potentially reorganize the phonological space in qualitatively different ways. With greater exposure to MAE, Heritage Mandarin performance was more L1-Mandarin-like, highlighting that accent familiarity is not a unitary mechanism, but interacts with native phonology and the dynamics of language learning.

## Figures and Tables

**Table 1:** Participant demographics obtained from the language background questionnaire. The median is provided for self-reported proficiency ratings. One standard deviation of the central tendency measure is presented in parentheses.

	Age of Acquisition (English)	English Proficiency		Chinese Proficiency		MAE Exposure	
		Listening	Speaking	Listening	Speaking	Familiarity	Exposure Likelihood
Native English Listeners (*n* = 22)	NA	10 (0.2)	10 (0)	NA	NA	4 (2.3)	2 (2)
Heritage Mandarin Listeners (*n* = 17)	2.5 (3)	10 (0.4)	10 (0.9)	8 (2.2)	7 (2.7)	8 (2.2)	6 (2.4)
L1-Mandarin Listeners (*n* = 26)	6.7 (2.3)	8 (1.1)	7 (1.3)	10 (0.4)	10 (0.2)	8 (1.2)	7 (2.1)

**Table 2:** Median acoustic measurements for the experimental stimuli by accent. Standard deviations are provided in parentheses.

	Overall Duration (ms)	Duration [s] (ms)	Center of Gravity [s] (Hz)	First Closure Duration (ms)	Voice Onset Time (ms)	Duration [a] (ms)	f0 (Hz)	F1 [a] (Hz)	F2 [a] (Hz)	Second Closure Duration (ms)	Duration [p]	Third Closure Duration (ms)	Duration [t] (ms)
CAE	657 (105)	151 (45)	7372 (1683)	44 (12)	19 (5)	156 (23)	188 (34)	845 (89)	1231 (101)	86 (37)	15 (31)	52 (16)	74 (27)
MAE	582 (81)	109 (21)	7025 (1563)	81 (51)	20 (9)	143 (22)	216 (26)	778 (112)	1211 (162)	65 (23)	21 (19)	65 (24)	73 (40)

## Data Availability

The data presented in this study are openly available in Open Science Framework at https://osf.io/8du7s/overview?view_only=864869e97b3b466487e378bbc1648c27 (The repository was created on 24 December 2025. It was last updated on 27 January 2026.).

## Supplementary Materials

The following supporting information can be downloaded at: https://www.mdpi.com/article/10.3390/brainsci16020174/s1, Table S1: Median ratings (*n* = 17) from the pre-test accentedness rating task for each of the ten talkers for each accent group selected to be included as items in the EEG experiment. Averaged medians and the standard deviation for each talker group are also presented. Talkers are listed according to recruitment order per accent; Figure S1: Distribution of the acoustic variables for CAE (pink) and MAE (blue). Individual points refer to individual talker values for each variable.
