# Supplementary material for: Language Experience Shapes Neural Grouping of Speech by Accent: EEG Evidence from Native, Second-Language, and Heritage Listeners
Source: Brain Sci. 2026 Jan 31;16(2):174. doi: 10.3390/brainsci16020174 (PMC12938406; doi:10.3390/brainsci16020174)
Supplement: Supplementary file 1 [file brainsci-16-00174-s001.zip › brainsci-4091534-supplementary.pdf]

Supplemental Materials

Talker	Median Rating	Standard Deviation
	Chinese Accented	
1	5	1.57
2	7	1.45
3	4	1.18
4	6	1.2
5	7	0.75
6	5	1.6
7	5	1.28
8	6	1.59
9	6	1.33
10	6	1.58
Overall	6	0.95 (median)
	Canadian English	
11	1	1.17
12	1	0.92
13	1	1.17
14	1	1.22
15	1	1.23
16	1	1.41
17	1	1.2
18	1	0.93
19	1	2.1
20	1	1.44
Overall	1	0 (median)

Table S1: Median ratings (n = 17) from the pre-test accentedness rating task for each of the ten talkers for each accent group selected to be included as items in the EEG experiment. Averaged medians and the standard deviation for each talker group are also presented. Talkers are listed according to recruitment order per accent.

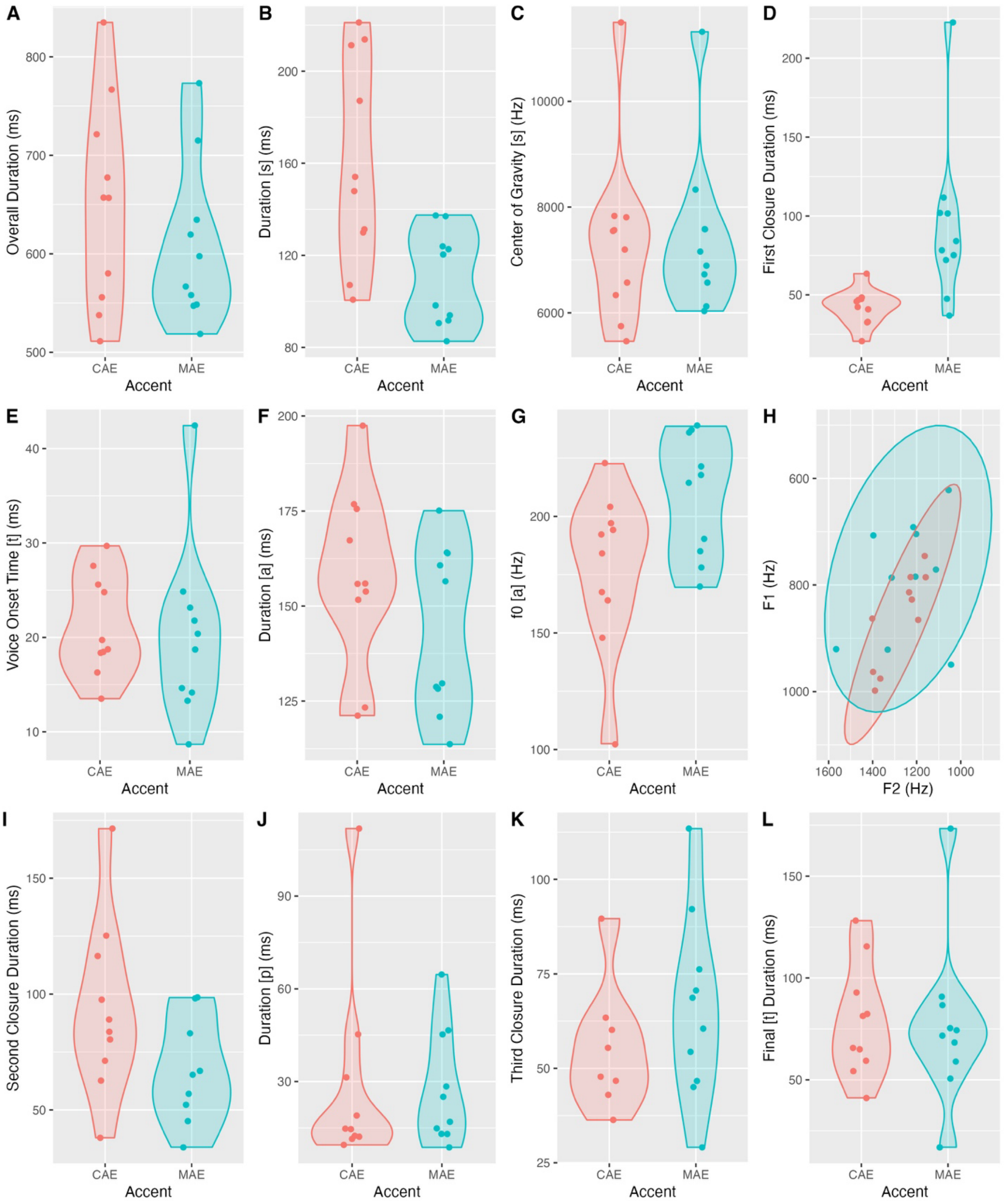

Figure S1: Distribution of the acoustic variables for CAE (pink) and MAE (blue). Individual points refer to individual talker values for each variable.
